# Contributions of park-based activities to overall physical activity among adults living near recently renovated parks in low-income New York City neighborhoods: variations by race/ethnicity and sex

**DOI:** 10.1186/s12966-025-01838-0

**Published:** 2025-11-12

**Authors:** Rachel L. Thompson, Luis David Olivera León, Houlin Hong, Justine Maffei, Katarzyna E. Wyka, Terry T.-K. Huang

**Affiliations:** Center for Systems and Community Design and NYU-CUNY Prevention Research Center, Graduate School of Public Health and Health Policy, City University of New York, 55 West 125th St, New York, NY 10027 USA

**Keywords:** Parks, Park use, Physical activity, Health disparities, Built environment, Urban health

## Abstract

**Background:**

Urban parks may promote physical activity (PA); however, little is known about whether *renovated* urban parks contribute to overall PA similarly across diverse sociodemographic groups. In this cross-sectional study, we examined associations between park-based activities and overall PA among adults living near recently renovated parks in low-income New York City (NYC) neighborhoods, with particular attention to differences by race/ethnicity and sex.

**Methods:**

A total of 1336 adult survey respondents who reported past-month use of a renovated park within 0.5 miles of their residence were included. Surveys captured past-month participation in activities at the renovated park (walking, exercising, taking children to the playground/park, relaxing, socializing, volunteering) and self-reported PA level during visits (sitting, light, moderate-to-vigorous). The outcome, overall past-week PA, was measured in metabolic equivalent of task (MET)-minutes using the International Physical Activity Questionnaire and log-transformed prior to analysis. Linear regression models estimated associations of park-based activities and park-based PA levels with log(MET-minutes) of overall PA in the full sample and stratified by race/ethnicity and sex.

**Results:**

In the full sample, park-based activities explained 5.4% of the variability in overall PA of past-month renovated park users; self-reported park-based PA level explained 5.2% of the variability. Compared to past-month park users who did not engage in these activities, those who reported exercising at the study park had 47% higher overall PA and those who reported walking had 33% higher overall PA, while those who reported volunteering had 26% lower overall PA, after confounder adjustment. Activities at renovated parks explained more variability in overall PA among males (6.3%) compared with females (5.1%), and among minority groups (Latino/as = 6.5%, Blacks = 6.8%, other race/ethnicity = 11.2%) compared with Whites (4.3%).

**Conclusions:**

Among adults with past-month renovated park use in low-income NYC neighborhoods, park-based activities explained a significant proportion of the variability in overall PA, with stronger contributions among minorities and males. These findings highlight the importance of considering sociodemographic differences when assessing the role of renovated urban parks in promoting PA and suggest that investments in high-quality green spaces may be particularly impactful for minority groups facing disproportionate barriers to PA.

**Supplementary Information:**

The online version contains supplementary material available at 10.1186/s12966-025-01838-0.

## Background

Regular physical activity (PA) is essential for preventing the onset of chronic and communicable disease, as well as premature death [1, 2]. Individuals who are insufficiently active have a 20 to 30% increased risk of death compared to those who are sufficiently active [2]. The World Health Organization reports that across the globe, 31% of adults and 80% of children fail to meet the recommended levels of weekly PA [2]. In the United States (US), racial minority groups are less likely to engage in sufficient PA compared to their White counterparts [3, 4]. According to the US Centers for Disease Control and Prevention, Non-Hispanic Black and Hispanic adults had the highest prevalence of physical inactivity nationwide [5]. Similarly, a study analyzing national data from the Canadian Community Health Survey found that among immigrants, disparities in PA existed only for those who also belonged to racial minority groups [6]. Evidence suggests that disparities in PA are likely rooted in structural inequities, rather than lifestyle choices [3, 6]. For example, racial minorities are more likely to live in neighborhoods with safety concerns, limited access to recreational facilities, and fewer structured activity options like organized sports and exercise programs [6].

Urban parks serve as free and open spaces for leisure-time PA, especially in low-income neighborhoods where access to private recreational opportunities, like gyms and fitness clubs, are limited [7, 8]. However, prior research has shown that parks in low-income neighborhoods are underutilized, and contribute less to overall PA than moderate- and high-income neighborhood parks [9]. This may result from a lack of programming and poorly maintained facilities in low-income neighborhood parks, which tend to attract fewer users [9].

Park quality improvement initiatives may be one means of improving the ability of urban parks to support PA in low-income neighborhoods. Parks considered to be of high quality typically have: (1) a variety of facilities and PA-supporting amenities (e.g., trails, playgrounds, ball courts), (2) well-maintained features and absence of litter or vandalism, (3) high accessibility and usability, (4) safety features (e.g., lighting, traffic management), and (5) comfort elements (e.g., bathrooms, drinking fountains, shade) [10]. We previously evaluated the association between PA and frequent use of urban parks that had been recently renovated to incorporate many of these features as a part of the Community Parks Initiative (CPI), a citywide park redesign and renovation program providing targeted improvements to neighborhood parks in disrepair situated in low-income New York City (NYC) neighborhoods [11]. Among a representative sample of 2000 adults living within a 0.5-mile radius of one of eight renovated parks, we found that individuals who reported frequent (≥ once/week) use of the renovated park had significantly higher PA levels than those who reported infrequent (< once/week) use of the park. Furthermore, this association was strongest among Latino/as and individuals in the lowest income group (<$25,000 annual household income). Other previous research has also linked elements of park quality, including perceived park quality, park cleanliness, park attractiveness, and presence of diverse park facilities, to higher PA and lower body mass index [12–15]. High quality parks, such as the renovated CPI parks in NYC, may therefore serve as more effective facilitators of PA in urban settings due to their PA-supporting amenities and overall appeal.

Although high-quality renovated parks may be promising facilitators of PA in low-income urban neighborhoods, their use and associated health benefits may vary across demographic groups, reflecting diverse cultural preferences and barriers to participation in park-based PA. Previous research has shown that different racial/ethnic groups and sexes interact with urban park spaces differently, with some groups participating in more PA-generating activities at parks than others [14, 16–21]. Findings on racial/ethnic differences in park use behaviors in the literature have been mixed, with several studies suggesting that White individuals are more likely to engage in PA at parks compared to racial/ethnic minority groups in the United States [14, 16, 17]. However, some studies have shown that Latino/as, specifically Spanish-speaking Latino/as, may use parks for exercise more often than other minority groups [17, 18], participating in a variety of activities at parks including socializing, passive leisure, and playing group sports like soccer [16, 19]. Park use behaviors and preferences also differ by sex. In a 2021 study, Cohen et al. showed that female park users were more likely than male park users to report bringing children to the park as the reason for their visit, while male park users were more likely to report relaxing as the reason for their park visit [20]. Furthermore, a review of 23 studies reporting on sex differences in observed park users showed that males consistently tended to visit parks more often than females, and that males were more frequently found engaging in PA at parks than females [21].

Differences in park use behaviors observed in past studies may reflect not only cultural differences and individual preferences, but also disparities in access and safety perceptions, as minority groups and women report higher barriers to park use, including concerns about poorly maintained facilities and safety [17, 22, 23]. High-quality renovated urban park spaces may address some of these barriers, but there remains a gap in our understanding of how park use behaviors differ in the context of high-quality urban park spaces, and the extent to which park-based PA contributes to overall PA across diverse sociodemographic groups.

In this study, we sought to examine how park-based activities and park-based PA at *high-quality*,* recently renovated* urban parks contribute to overall PA across diverse sociodemographic groups. Building on our previous research in low-income NYC neighborhoods served by recently renovated CPI parks [11], we analyzed differences in park-based activities and PA levels by race/ethnicity and sex among individuals who visited their renovated study park in the past month. Additionally, we evaluated the degree to which park-based activities contributed to overall PA levels and explored variations in this contribution by race/ethnicity and sex. This analysis offers valuable insights into the extent to which activities at high-quality urban parks contribute to overall PA across diverse populations.

## Methods

This study follows the STROBE guidelines for reporting cross-sectional observational studies (Additional File 1) [24].

### Setting

Starting in 2014, the CPI is an ongoing equity-focused program aimed at redesigning and renovating urban parks in low-income, diverse neighborhoods across NYC [25]. Since its inception, the CPI has led to the renovation of 67 neighborhood parks, with another 50 projects actively underway as of March 2025 [26]. The present analysis used cross-sectional survey data collected at baseline as part of the Supporting Parks and Revitalizing Communities Study (SPARCS) – a community-engaged intervention study conducted across eight recently renovated CPI sites in NYC [27]. SPARCS study sites included two sites in each of the four largest boroughs in NYC: Manhattan, Brooklyn, Queens, and the Bronx. The eight sites included in this study received comprehensive redesigns and renovations which were completed between June 2016 and April 2021 with a median capital investment of US $5.2 million [range: $3.0 million - $11.7 million] [28]. Renovations were tailored for each site based on community input [29], but generally incorporated many quality enhancements such as landscaping, seating and shade structures, adult fitness equipment, ball courts, playgrounds, picnic areas and expanded green spaces [30]. In addition to prior CPI renovations, the eight included parks were selected based on pre-established community partnerships and sufficient population size (*n* ≥ 20,000 adult contacts in each neighborhood, given that a 1–2% response rate is expected in polling research).

### Survey data collection

Participants were recruited from the residential areas located within a 0.5-mile radius of each of the eight included CPI parks. Recruitment was conducted by the Consensus Strategies polling firm (Waltham, MA) via email and text message. A stratified random sampling approach was employed with a minimum of 25 participants per stratum per park catchment area. Stratification was based on sex, age, race/ethnicity, and education level, with enrollment targets chosen to reflect the demographic composition of each park’s surrounding neighborhood. A total of 2067 participants completed the survey in June 2023 (baseline of SPARCS). The survey was available in English, Spanish, and Chinese, with translations verified by SPARCS research team members fluent in these languages.

### Measures

#### Outcome – MET-minutes of PA

The primary outcome for this analysis was past seven-day overall PA in metabolic equivalent of task (MET)-minutes. MET-minutes of PA were captured using the validated seven-item International Physical Activity Questionnaire (IPAQ) [31]. The IPAQ instrument characterizes respondent PA in the past week through questions about activity types (i.e., walking, moderate intensity activity, and vigorous intensity activity) and the frequency and duration of those activities in the past week. Walking activity was defined as any walking done at work or home, walking to travel from place to place, and any other walking done solely for recreation. Moderate intensity activity was defined as any activity taking moderate physical effort that made one breathe somewhat harder than normal, such as carrying light loads or bicycling at a regular pace. Vigorous intensity activity was defined as any activity taking hard physical effort that made one breathe much harder than normal, such as heavy lifting or aerobics. MET-minutes per week for each activity type was found by multiplying a constant MET value (walking = 3.3, moderate activity = 4, vigorous activity = 8) by the minutes spent in that activity on a usual day and again by the number of days per week spent performing that activity. Total MET-minutes per week was calculated as the sum of walking MET-minutes, moderate MET-minutes, and vigorous MET-minutes.

#### Past-month park use, park-based activities, and park-based PA

Past-month study park use and engagement in park-based activities were assessed using validated survey items from adapted from Veitch et al. [32]. Past-month study park use was determined based on response to the question “In the past 30 days, on average, how often have you visited the [study park]?” with options (1) daily, (2) 4–6 times per week, (3) 2–3 times per week, (4) once per week, (5) 2–3 times per month, (6) once per month, (7) less than once per month, or (8) have not visited in the past 30 days. Participants who responded with any option other than (8) (have not visited in the past 30 days) were considered to be past-month study park users, and were prompted with the following question to assess participation in park-based activities: “In the past 30 days, what activities did you usually do during your visits to [study park]?” with the option of responding “Yes” or “No” to a variety of specific activities that we subsequently organized into six activity groups: (1) walked (including those who went for a recreational walk, walked the dog, or reported going from place-to-place through the park), (2) exercised (including those who went for a jog/run, rode a bike, played ball games, or did other exercise at the park), (3) took children to the playground/park, (4) relaxed (including those who reported that they relaxed or viewed nature), (5) socialized (including those who had a picnic/BBQ, socialized with family/friends, attended a private event/celebration/birthday, or attended a public event or program), and (6) volunteered (including those who volunteered in park maintenance or other programs). An additional question assessed usual PA level during past-month study park visits: “In the past 30 days, which of the following best describes your usual physical activity level during your visits to [study park]?”, with options “Mostly sitting”, “Mostly light activities”, “Mostly moderate activities”, or “Mostly vigorous activities”. Due to a low number of responses to the “Mostly vigorous activities” option, a combined category of “Mostly moderate-to-vigorous activities” was created for analysis.

#### Race/ethnicity

A composite race/ethnicity variable was created based on self-identified race and Latino/a ethnicity. Any person responding “Yes” to the question “Are you Hispanic or Latino?” was considered Latino/a. Remaining individuals were classified in one of three groups: non-Latino/a White, non-Latino/a Black and other. The “other” group was created as a combination of less common racial identities to facilitate robust statistical analyses and included individuals who identified as American Indian/Alaskan Native, Asian/Pacific Islander, Middle Eastern/North African, or two or more races.

#### Other sociodemographic variables and covariates

Other sociodemographic variables included: self-identified sex (male, female), education level (some college or more, high school graduate or less), annual household income (<$25,000, $25,000-$75,000, ≥$75,000), age (18-35y, 36-59y, ≥ 60y), and employment status (employed or self-employed, retired, not employed). Frequency of study park use and frequency of use of other parks in the neighborhood were also used as covariates, with values “Less than once per week” and “Once per week or more”.

### Statistical analysis

For this study, the overall sample was restricted to include only survey respondents with past 30-day study park use. Descriptive statistics (n (%) and median [IQR]) were generated for all sociodemographic variables and MET-minutes of PA. Chi-squared tests were used to compare participation in park-based activities and self-reported usual PA level at the CPI study park by race/ethnicity and sex.

Linear regression models were used to characterize the contribution of participation in park-based activities to overall PA in the overall sample of past-month study park users and separately by race/ethnicity, sex, and the combination of race/ethnicity and sex. Prior to analysis, the outcome variable (MET-minutes of PA) was natural log-transformed after adding a small amount (+ 30 min) to each observation to account for zero values. Unadjusted models included the six binary indicators of park-based activities (walked, exercised, took children to playground/park, relaxed, socialized, and volunteered). Adjusted models additionally included education level, annual household income, age, employment status, frequency of study park use, frequency of other park use, and study site as covariates. From unadjusted models, R^2^ values were obtained to quantify the variance in overall PA explained by park-based activity variables. From both the unadjusted and adjusted models, beta values and 95% confidence intervals were exponentiated to characterize the multiplicative difference in MET-minutes of PA among survey respondents who engaged in a given park-based activity compared to those who did not engage in that activity. Additional unadjusted and adjusted linear regression models using usual PA level during visits to the study park in the past 30 days were also fit to the sample overall and for each race/ethnicity and sex combination. Assumptions for linear regression were verified using residual plots, and variance inflation factors (VIF) were checked to confirm the absence of multicollinearity (VIF < 2).

Statistical analyses were performed using SAS software, Version 9.4 (SAS Institute Inc., Cary, NC, USA). Linear regression modeling was performed using PROC GLM, and VIF values were obtained using PROC REG. Statistical significance was defined as α = 0.05 for all analyses.

## Results

### Sample sociodemographic characteristics

Out of 2,067 individuals who completed the survey, 1336 who reported using their renovated study park at least once in the past 30 days were included in this analysis. The sample of survey respondents with past 30-day study park use was predominantly Latino/a or Black, with 36% identifying as Latino/a, 28% as non-Latino/a Black, 22% as non-Latino/a White, and 14% as another race/ethnicity (Table 1). Survey respondents were majority female (65%) with at least some college education (77%), and predominantly lower-to-middle annual household income (24% with <$25,000, 38% with $25,000-$75,000, and 38% with ≥$75,000). Thirty-three percent of respondents were aged 18-35y, 53% were aged 36-59y, and 14% were aged ≥ 60y. Sixty-seven percent of the sample was employed or self-employed. The sample contained roughly equal representation (~ 25%) of residents from each of the four participating boroughs (Bronx, Brooklyn, Manhattan, and Queens). Compared to survey respondents with no past-month study park use, those with past-month study park use were more likely to be Latino/a, male, and under 60 years old (Additional File 2- Supplemental Table 1). Furthermore, MET-minutes of PA were significantly higher among past-month study park users (median [IQR] = 1386 [584-2853]) compared to non-users (median [IQR] = 1173 [396-2772]; *p* = 0.001; Additional File 2 - Supplemental Table 1).

**Table 1:** Characteristics of past 30-day study park users living within a 0.5-mile radius of a recently renovated park in NYC

	Visited study park in the past 30 days *n* = 1336^a^
Race/Ethnicity	
Latino/a	475 (36%)
Non-Latino/a Black	378 (28%)
Non-Latino/a White	291 (22%)
Other	192 (14%)
Sex	
Female	864 (65%)
Male	472 (35%)
Education level	
Some college or more	1028 (77%)
High school graduate or less	308 (23%)
Annual household income	
<$25,000	321 (24%)
$25,000-$75,000	510 (38%)
≥$75,000	505 (38%)
Age	
18-35y	441 (33%)
36-59y	705 (53%)
>60y	190 (14%)
Employment status	
Employed or self-employed	898 (67%)
Not employed	316 (24%)
Retired	122 (9%)
Borough	
Bronx	319 (24%)
Brooklyn	328 (25%)
Manhattan	330 (25%)
Queens	359 (27%)
MET-minutes of physical activity	
	1386 [584-2853]

*Abbreviations*: *CPI* Community Parks Initiative, *NYC* New York City

^a^n (%); median [IQR]

### Differences in park-based activities and park-based PA by race/ethnicity and sex among past-month renovated park users

Among all survey respondents with past-month study park use, walking was the most frequently reported activity (88%), followed by relaxing (79%), socializing (71%), exercising (66%), taking children to the playground/park (59%), and volunteering (18%) (Table 2). There were significant differences across racial/ethnic groups in taking children to the playground/park (χ^2^ = 14.74; *p* = 0.002) and socializing (χ^2^ = 8.09; *p* = 0.044) at the study park in the past 30 days. Sixty-three percent of Latino/as and 64% of non-Latino/a Blacks reported taking children to the playground/park compared to only 53% of non-Latino/a Whites and 52% of those of other races/ethnicities. Similarly, 75% of Latino/as and 71% of non-Latino Blacks reported socializing at the study park, compared to only 66% of non-Latino Whites and 68% of those with other races/ethnicities. There were no differences in self-reported usual PA level during visits to the study park in the past 30 days by race/ethnicity (χ^2^ = 7.85; *p* = 0.249).

**Table 2:** Participation in park-based activities and self-reported usual PA level during visits to study park in the past 30 days for overall survey sample and by race/ethnicity

	Overall *n* = 1336	Latino/a *n* = 475	Non-Latino/a Black *n* = 378	Non-Latino/a White *n* = 291	Other *n* = 192	χ^2^	*p*-value
*Activities usually performed during visits to study park in past 30 days*							
Walked	1175 (88%)	428 (90%)	325 (86%)	260 (89%)	162 (84%)	6.32	0.097
Exercised	884 (66%)	332 (70%)	245 (65%)	181 (62%)	126 (66%)	5.33	0.149
Took children to playground/park	794 (59%)	300 (63%)	240 (64%)	154 (53%)	100 (52%)	14.74	0.002
Relaxed	1061 (79%)	383 (81%)	304 (80%)	226 (78%)	148 (77%)	1.85	0.604
Socialized	947 (71%)	357 (75%)	267 (71%)	193 (66%)	130 (68%)	8.09	0.044
Volunteered	235 (18%)	85 (18%)	72 (19%)	46 (16%)	32 (17%)	1.34	0.721
*Usual physical activity level during visits to study park in past 30 days*							
Mostly moderate-to-vigorous activities	376 (28%)	146 (31%)	109 (29%)	80 (27%)	41 (21%)	7.85	0.249
Mostly light activities	564 (42%)	189 (40%)	154 (41%)	132 (45%)	89 (46%)		
Mostly sitting activities	396 (30%)	140 (29%)	115 (30%)	79 (27%)	62 (32%)		

There were also significant differences in usual park activities by sex (Table 3). Males were more likely to report exercising (75%) at the study park than females (62%) (χ^2^ = 23.05; *p* < 0.001), whereas females were more likely to report taking children to the playground/park (64%) than males (51%) (χ^2^ = 22.30; *p* < 0.001). Males also more commonly reported volunteering at the study park (24%) compared to females (14%) (χ^2^ = 21.69; *p* < 0.001). However, there were no significant differences in self-reported usual PA level during visits to the study park in the past 30 days by sex (χ^2^ = 4.74; *p* = 0.094).

Differences by sex in usual park activities exhibited similar trends across most racial/ethnic groups (Table 3). Latino and non-Latino White males were more likely to report exercising in the study park than their female counterparts (Latino/a χ^2^ = 15.60, *p* < 0.001; non-Latino/a White χ^2^ = 13.03, *p* = < 0.001). Similarly, Latino, non-Latino Black, and non-Latino White males all more commonly reported volunteering in the study park compared to females (Latino/a χ^2^ = 6.30, *p* = 0.012; non-Latino/a Black χ^2^ = 6.40, *p* = 0.011; non-Latino/a White χ^2^ = 17.13, *p* < 0.001). Latina and non-Latina Black females were more likely to report taking children to the playground/park than their male counterparts (Latino/a χ^2^ = 9.33, *p* = 0.002; non-Latino/a Black χ^2^ = 16.28, *p* < 0.001). Within the other racial/ethnic group, there were no significant differences in any reported park activities by sex.

Self-reported usual PA level during visits to the study park in the past 30 days significantly differed by sex among non-Latino/a Blacks (χ^2^ = 14.53; *p* < 0.001) and Whites (χ^2^ = 10.04; *p* = 0.007), but not among Latino/as (χ^2^ = 2.31; *p* = 0.315) or other races/ethnicities (χ^2^ = 2.97; *p* = 0.227; Table 3).

**Table 3:** Participation in park-based activities and self-reported usual PA level during visits to study park in the past 30 days by sex in the full sample and within each race/ethnicity

	Female	Male	χ^2^	*p*-value
**Overall**	*n* = 864	*n* = 472		
*Activities usually performed during visits to study park in past 30 days*				
Walked	762 (88%)	413 (88%)	0.14	0.709
Exercised	532 (62%)	352 (75%)	23.05	< 0.001
Took children to playground/park	554 (64%)	240 (51%)	22.30	< 0.001
Relaxed	690 (80%)	371 (79%)	0.30	0.586
Socialized	621 (72%)	326 (69%)	1.17	0.280
Volunteered	121 (14%)	114 (24%)	21.69	< 0.001
*Usual physical activity level during visits to study park in past 30 days*				
Mostly moderate-to-vigorous activities	228 (26%)	148 (31%)	4.74	0.094
Mostly light activities	381 (44%)	183 (39%)		
Mostly sitting activities	255 (30%)	141 (30%)		
**Latino/a**	*n* = 328	*n* = 147		
*Activities usually performed during visits to study park in past 30 days*				
Walked	300 (91%)	128 (87%)	2.19	0.139
Exercised	211 (64%)	121 (82%)	15.60	< 0.001
Took children to playground/park	222 (68%)	78 (53%)	9.33	0.002
Relaxed	267 (81%)	116 (79%)	0.40	0.525
Socialized	254 (77%)	103 (70%)	2.95	0.086
Volunteered	49 (15%)	36 (24%)	6.30	0.012
*Usual physical activity level during visits to study park in past 30 days*				
Mostly moderate-to-vigorous activities	107 (33%)	39 (27%)	2.31	0.315
Mostly light activities	130 (40%)	59 (40%)		
Mostly sitting activities	91 (28%)	49 (33%)		
**Non-Latino/a Black**	*n* = 276	*n* = 102		
*Activities usually performed during visits to study park in past 30 days*				
Walked	240 (87%)	85 (83%)	0.81	0.368
Exercised	172 (62%)	73 (72%)	2.79	0.095
Took children to playground/park	192 (70%)	48 (47%)	16.28	< 0.001
Relaxed	222 (80%)	82 (80%)	0.00	0.993
Socialized	193 (70%)	74 (73%)	0.25	0.619
Volunteered	44 (16%)	28 (27%)	6.40	0.011
*Usual physical activity level during visits to study park in past 30 days*				
Mostly moderate-to-vigorous activities	67 (24%)	42 (41%)	14.53	< 0.001
Mostly light activities	127 (46%)	27 (26%)		
Mostly sitting activities	82 (30%)	33 (32%)		
**Non-Latino/a White**	*n* = 151	*n* = 140		
*Activities usually performed during visits to study park in past 30 days*				
Walked	134 (89%)	126 (90%)	0.12	0.728
Exercised	79 (52%)	102 (73%)	13.03	< 0.001
Took children to playground/park	83 (55%)	71 (51%)	0.53	0.468
Relaxed	116 (77%)	110 (79%)	0.13	0.720
Socialized	100 (66%)	93 (66%)	0.00	0.971
Volunteered	11 (7%)	35 (25%)	17.13	< 0.001
*Usual physical activity level during visits to study park in past 30 days*				
Mostly moderate-to-vigorous activities	30 (20%)	50 (36%)	10.04	0.007
Mostly light activities	79 (52%)	53 (38%)		
Mostly sitting activities	42 (28%)	37 (26%)		
**Other**	*n* = 109	*n* = 83		
*Activities usually performed during visits to study park in past 30 days*				
Walked	88 (81%)	74 (89%)	2.54	0.111
Exercised	70 (64%)	56 (67%)	0.22	0.639
Took children to playground/park	57 (52%)	43 (52%)	0.00	0.947
Relaxed	85 (78%)	63 (76%)	0.12	0.734
Socialized	74 (68%)	56 (67%)	0.00	0.951
Volunteered	17 (16%)	15 (18%)	0.21	0.648
*Usual physical activity level during visits to study park in past 30 days*				
Mostly moderate-to-vigorous activities	24 (22%)	17 (20%)	2.97	0.227
Mostly light activities	45 (41%)	44 (53%)		
Mostly sitting activities	40 (37%)	22 (27%)		

### Contributions of park-based activities to overall PA among past-month renovated park users

In the overall sample of past-month renovated park users, park-based activities explained 5.4% of the variability in overall PA (Table 4). After adjusting for covariates, exercising showed the strongest association with overall PA, where those who had exercised at the study park had 47% higher MET-minutes of overall PA compared to those who had not exercised (adjusted e^β^ = 1.47; 95% CI 1.26, 1.72). Walking was also a positive correlate of overall PA, with individuals who walked exhibiting 33% higher MET-minutes of PA than those who did not walk (adjusted e^β^ = 1.33; 95% CI 1.09, 1.64). Volunteering had a negative association with overall PA, with those who volunteered at the study park having 26% lower MET-minutes of PA compared to those who did not volunteer (adjusted e^β^ = 0.74; 95% CI 0.61, 0.89).

### Differences in contributions of park-based activities to overall PA among past-month renovated park users by race/ethnicity and sex

Analyses by race/ethnicity revealed differences in the contributions of park-based activities to overall PA, including the amount of variability in overall PA explained by park-based activities, as well as the contribution of specific activities to overall PA (Table 4). Among Latino/as, activities at renovated parks explained 6.5% of the variability in overall PA; both exercising (adjusted e^β^ = 1.54; 95% CI 1.17, 2.03) and socializing (adjusted e^β^ = 1.41; 95% CI 1.05, 1.90) were positively associated with overall PA, after adjusting for covariates. For non-Latino/a Blacks, park-based activities explained 6.8% of the variability, with walking (adjusted e^β^ = 1.49; 95% CI 1.00, 2.21) and exercising (adjusted e^β^ = 1.38; 95% CI 1.01, 1.89) emerging as positive correlates of overall PA. Among non-Latino/a Whites, park-based activities explained only 4.3% of the variability in overall PA; no activity was significantly positively associated with overall PA after covariate adjustment, while taking children to the playground/park was significantly negatively associated with overall PA (adjusted e^β^ = 0.73; 95% CI 0.55, 0.98). For individuals with other races/ethnicities, park-based activities explained 11.2% of the variability in overall PA, with taking children to the playground/park being the sole activity positively associated with overall PA after covariate adjustment (adjusted e^β^ = 1.64; 95% CI 1.11, 2.43).

**Table 4:** Park-based activities as correlates of MET-minutes of PA in the overall survey sample and by race/ethnicity

Subgroup	Park-Based Activity	Unadjusted Models			Adjusted Models^b^	
		e^β^ (95% CI)^a^	*p*-value	Model *R*^2^	e^β^ (95% CI)^a^	*p*-value
**Overall** (*n* = 1336)	Walked	1.38 (1.12, 1.71)	0.003	0.054	1.33 (1.09, 1.64)	0.006
	Exercised	1.53 (1.31, 1.78)	< 0.001		1.47 (1.26, 1.72)	< 0.001
	Took children to playground/park	0.97 (0.84, 1.12)	0.693		1.03 (0.89, 1.19)	0.699
	Relaxed	1.11 (0.93, 1.32)	0.241		1.18 (0.99, 1.40)	0.063
	Socialized	1.19 (1.00, 1.40)	0.045		1.15 (0.98, 1.36)	0.092
	Volunteered	0.70 (0.58, 0.84)	< 0.001		0.74 (0.61, 0.89)	0.001
**Latino/a** (*n* = 475)	Walked	1.22 (0.82, 1.82)	0.324	0.065	1.24 (0.84, 1.85)	0.280
	Exercised	1.65 (1.26, 2.15)	< 0.001		1.54 (1.17, 2.03)	0.002
	Took children to playground/park	0.95 (0.74, 1.21)	0.681		1.00 (0.78, 1.29)	0.972
	Relaxed	1.06 (0.77, 1.44)	0.731		1.13 (0.83, 1.54)	0.449
	Socialized	1.42 (1.05, 1.91)	0.021		1.41 (1.05, 1.90)	0.023
	Volunteered	0.77 (0.57, 1.04)	0.085		0.80 (0.59, 1.09)	0.155
**Non-Latino/a Black** (*n* = 378)	Walked	1.52 (1.03, 2.23)	0.034	0.068	1.49 (1.00, 2.21)	0.049
	Exercised	1.48 (1.09, 2.00)	0.011		1.38 (1.01, 1.89)	0.044
	Took children to playground/park	1.02 (0.77, 1.34)	0.890		1.09 (0.80, 1.48)	0.572
	Relaxed	1.28 (0.90, 1.81)	0.170		1.41 (0.98, 2.04)	0.066
	Socialized	1.13 (0.82, 1.56)	0.451		1.10 (0.78, 1.53)	0.593
	Volunteered	0.66 (0.47, 0.94)	0.020		0.71 (0.49, 1.03)	0.073
**Non-Latino/a White** (*n* = 291)	Walked	1.11 (0.72, 1.70)	0.628	0.043	1.12 (0.73, 1.73)	0.590
	Exercised	1.37 (1.03, 1.82)	0.032		1.29 (0.96, 1.75)	0.096
	Took children to playground/park	0.76 (0.58, 0.99)	0.046		0.73 (0.55, 0.98)	0.038
	Relaxed	1.14 (0.82, 1.58)	0.429		1.21 (0.86, 1.68)	0.268
	Socialized	1.01 (0.74, 1.38)	0.946		0.95 (0.69, 1.30)	0.733
	Volunteered	0.73 (0.50, 1.05)	0.088		0.85 (0.56, 1.30)	0.459
**Other** (*n* = 192)	Walked	1.38 (0.84, 2.27)	0.206	0.112	1.60 (0.96, 2.66)	0.071
	Exercised	1.67 (1.09, 2.57)	0.019		1.39 (0.90, 2.15)	0.132
	Took children to playground/park	1.45 (0.99, 2.12)	0.053		1.64 (1.11, 2.43)	0.013
	Relaxed	0.95 (0.61, 1.48)	0.830		0.98 (0.63, 1.52)	0.922
	Socialized	1.25 (0.79, 1.98)	0.330		1.25 (0.79, 1.97)	0.332
	Volunteered	0.64 (0.39, 1.05)	0.078		0.67 (0.41, 1.10)	0.114

*Abbreviations*: *PA* Physical activity, *MET* Metabolic equivalent of task

^a^Obtained from linear regression models with the outcome log(MET-minutes of past-week PA + 30)

^b^Additionally adjusted for sex, education level, annual household income, age, employment status, frequency of study park use, frequency of other park use, and study site. The overall model was also adjusted for race/ethnicity

Analyses by sex revealed some small differences in the contribution of park-based activities to overall PA by sex (Table 5). For females, park-based activities explained 5.1% of the variability in overall PA, with walking (adjusted e^β^ = 1.30; 95% CI 1.00, 1.69) and exercising (adjusted e^β^ = 1.46; 95% CI 1.22, 1.76), acting as positive correlates of overall PA, and volunteering (adjusted e^β^ = 0.73; 95% CI 0.57, 0.94) acting as a negative correlate. For males, park-based activities explained 6.3% of the variability in overall PA, with walking (adjusted e^β^ = 1.43; 95% CI 1.00, 2.04) and exercising (adjusted e^β^ = 1.54; 95% CI 1.14, 2.07), also acting as positive correlates of overall PA. When looking across racial/ethnic groups, park-based activities explained the greatest amount of variability in the overall PA of Latino males (10.0%), non-Latino Black males (11.3%), other females (12.9%), and other males (12.8%). Park-based activities explained the smallest amount of variability in the overall PA of non-Latina White females (5.7%) and non-Latino White males (4.5%).

**Table 5:** Park-based activities as correlates of MET-minutes of PA by sex and race/ethnicity

Subgroup	Park-Based Activity	Unadjusted Models			Adjusted Models^b^	
		e^β^ (95% CI)^a^	*p*-value	Model *R*^2^	e^β^ (95% CI)^a^	*p*-value
**Female** (*n* = 864)	Walked	1.32 (1.01, 1.71)	0.040	0.051	1.30 (1.00, 1.69)	0.048
	Exercised	1.46 (1.21, 1.76)	< 0.001		1.46 (1.22, 1.76)	< 0.001
	Took children to playground/park	0.95 (0.80, 1.13)	0.568		1.01 (0.84, 1.22)	0.881
	Relaxed	1.05 (0.84, 1.30)	0.674		1.12 (0.90, 1.39)	0.311
	Socialized	1.27 (1.04, 1.56)	0.022		1.18 (0.96, 1.44)	0.116
	Volunteered	0.67 (0.53, 0.86)	0.001		0.73 (0.57, 0.94)	0.015
**Male** (*n* = 472)	Walked	1.53 (1.07, 2.20)	0.020	0.063	1.43 (1.00, 2.04)	0.048
	Exercised	1.54 (1.15, 2.07)	0.004		1.54 (1.14, 2.07)	0.005
	Took children to playground/park	1.11 (0.86, 1.44)	0.414		1.04 (0.80, 1.35)	0.759
	Relaxed	1.24 (0.92, 1.66)	0.154		1.27 (0.95, 1.71)	0.110
	Socialized	1.06 (0.79, 1.43)	0.683		1.10 (0.82, 1.48)	0.514
	Volunteered	0.67 (0.51, 0.90)	0.007		0.77 (0.57, 1.03)	0.075
**Latina Female** (*n* = 328)	Walked	0.87 (0.53, 1.42)	0.567	0.071	0.96 (0.58, 1.58)	0.873
	Exercised	1.75 (1.29, 2.37)	< 0.001		1.63 (1.19, 2.23)	0.002
	Took children to playground/park	0.87 (0.65, 1.17)	0.363		1.01 (0.74, 1.38)	0.942
	Relaxed	0.97 (0.67, 1.40)	0.883		0.98 (0.68, 1.42)	0.916
	Socialized	1.49 (1.05, 2.11)	0.025		1.40 (0.98, 2.00)	0.065
	Volunteered	0.74 (0.51, 1.08)	0.122		0.74 (0.50, 1.10)	0.135
**Latino Male** (*n* = 147)	Walked	2.16 (1.07, 4.36)	0.032	0.100	1.86 (0.92, 3.76)	0.082
	Exercised	1.57 (0.85, 2.89)	0.146		1.75 (0.93, 3.28)	0.082
	Took children to playground/park	1.26 (0.76, 2.09)	0.361		0.99 (0.59, 1.65)	0.957
	Relaxed	1.26 (0.70, 2.25)	0.437		1.57 (0.83, 2.97)	0.162
	Socialized	1.09 (0.60, 1.97)	0.785		1.20 (0.65, 2.21)	0.548
	Volunteered	0.78 (0.45, 1.34)	0.364		0.98 (0.55, 1.74)	0.949
**Non-Latina Black Female** (*n* = 276)	Walked	1.57 (1.00, 2.47)	0.050	0.064	1.56 (0.98, 2.48)	0.062
	Exercised	1.41 (1.00, 1.97)	0.047		1.27 (0.90, 1.81)	0.177
	Took children to playground/park	1.01 (0.73, 1.42)	0.938		0.99 (0.69, 1.43)	0.971
	Relaxed	1.08 (0.72, 1.60)	0.712		1.12 (0.72, 1.73)	0.613
	Socialized	1.30 (0.90, 1.86)	0.163		1.31 (0.89, 1.92)	0.173
	Volunteered	0.64 (0.42, 0.98)	0.040		0.69 (0.44, 1.09)	0.115
**Non-Latino Black Male** (*n* = 102)	Walked	1.41 (0.63, 3.18)	0.397	0.113	1.50 (0.60, 3.73)	0.377
	Exercised	1.65 (0.77, 3.55)	0.195		1.64 (0.69, 3.86)	0.256
	Took children to playground/park	1.12 (0.61, 2.06)	0.709		1.03 (0.53, 2.00)	0.924
	Relaxed	1.96 (0.94, 4.08)	0.070		2.48 (1.14, 5.40)	0.023
	Socialized	0.77 (0.39, 1.54)	0.463		0.68 (0.31, 1.49)	0.335
	Volunteered	0.66 (0.35, 1.25)	0.201		0.85 (0.42, 1.72)	0.641
**Non-Latina White Female** (*n* = 151)	Walked	1.27 (0.68, 2.39)	0.447	0.057	1.39 (0.72, 2.67)	0.324
	Exercised	1.28 (0.87, 1.89)	0.214		1.39 (0.91, 2.11)	0.124
	Took children to playground/park	0.70 (0.48, 1.03)	0.070		0.69 (0.44, 1.06)	0.092
	Relaxed	1.21 (0.75, 1.96)	0.438		1.60 (0.96, 2.68)	0.073
	Socialized	0.96 (0.61, 1.52)	0.875		0.75 (0.45, 1.23)	0.248
	Volunteered	0.88 (0.43, 1.80)	0.716		1.41 (0.62, 3.21)	0.408
**Non-Latino White Male** (*n* = 140)	Walked	0.91 (0.50, 1.66)	0.757	0.045	0.90 (0.47, 1.73)	0.758
	Exercised	1.34 (0.86, 2.09)	0.190		1.26 (0.78, 2.03)	0.348
	Took children to playground/park	0.92 (0.61, 1.39)	0.677		0.88 (0.56, 1.37)	0.560
	Relaxed	1.06 (0.68, 1.66)	0.781		1.06 (0.66, 1.72)	0.798
	Socialized	1.01 (0.64, 1.59)	0.972		1.01 (0.63, 1.59)	0.980
	Volunteered	0.61 (0.39, 0.95)	0.030		0.72 (0.43, 1.22)	0.221
**Other Female** (*n* = 109)	Walked	1.32 (0.73, 2.41)	0.356	0.129	1.79 (0.95, 3.38)	0.071
	Exercised	1.47 (0.85, 2.55)	0.166		1.24 (0.71, 2.18)	0.439
	Took children to playground/park	1.82 (1.14, 2.92)	0.013		2.39 (1.40, 4.08)	0.002
	Relaxed	1.16 (0.64, 2.09)	0.630		1.10 (0.61, 1.99)	0.741
	Socialized	1.11 (0.63, 1.96)	0.710		1.27 (0.70, 2.29)	0.424
	Volunteered	0.61 (0.31, 1.17)	0.137		0.61 (0.31, 1.20)	0.150
**Other Male** (*n* = 83)	Walked	1.48 (0.57, 3.85)	0.420	0.128	1.59 (0.60, 4.22)	0.343
	Exercised	1.86 (0.91, 3.78)	0.088		1.42 (0.66, 3.06)	0.371
	Took children to playground/park	0.94 (0.48, 1.87)	0.866		1.19 (0.57, 2.48)	0.645
	Relaxed	0.79 (0.40, 1.58)	0.505		1.01 (0.49, 2.09)	0.968
	Socialized	1.82 (0.80, 4.15)	0.152		1.54 (0.64, 3.73)	0.329
	Volunteered	0.65 (0.30, 1.42)	0.275		0.66 (0.29, 1.49)	0.311

*Abbreviations*: *PA* Physical activity, *MET* Metabolic equivalent of task

^a^Obtained from linear regression models with the outcome log(MET-minutes of past-week PA + 30)

^b^Additionally adjusted for education level, annual household income, age, employment status, frequency of study park use, frequency of other park use, and study site. The overall models for males and females were also adjusted for race/ethnicity

### Contributions of self-reported usual park-based PA level to overall PA among past-month renovated park users

Self-reported usual park-based PA levels explained 5.2% of the variability in overall PA in the overall sample of past-month renovated park users (Additional File 2 - Supplemental Table 2). Compared to those reporting mostly sitting activities at the study park, individuals who reported mostly moderate-to-vigorous activities had 95% higher overall PA (adjusted e^β^ = 1.95; 95% CI 1.64, 2.32), and individuals who reported mostly light activities had 51% higher overall PA (adjusted e^β^ = 1.51; 95% CI 1.29, 1.76). Self-reported park-based PA generally explained a greater proportion of the variability in overall PA among Latino/as (R^2^ = 5.3%), non-Latino/a Blacks (R^2^ = 5.7%) and other races/ethnicities (R^2^ = 8.4%) compared to Non-Latino/a Whites (R^2^ = 2.6%) (Additional File 2 - Supplemental Table 2), and among males (R^2^ = 7.5%) compared to females (R^2^ = 4.1%) (Additional File 2 - Supplemental Table 3). Among race/ethnicity and sex combinations, the highest amount of variability in overall PA was explained by usual park-based PA levels among non-Latino Black males (R^2^ = 13.7%), other females (R^2^ = 10.7%), and Latino males (R^2^ = 8.1%) (Additional File 2 - Supplemental Table 3). The lowest amount of variability in overall PA explained by self-reported park-based PA was observed among non-Latina White females (R^2^ = 1.5%) and non-Latina Black females (R^2^ = 3.1%).

## Discussion

In a large and diverse sample of adults from low-income NYC neighborhoods who reported past-month renovated park use, we found that park-based activities and park-based PA levels were significant contributors to overall PA, with racial/ethnic minority groups and males showing the strongest associations. Exercising and walking in renovated park spaces emerged as consistent positive correlates of overall PA across multiple demographic groups. Additionally, park-based activities explained a greater proportion of variability in overall PA among males compared to females, and among Latino/as, non-Latino/a Black individuals, and those of other races/ethnicities compared to non-Latino/a Whites. To our knowledge, this is the first study to evaluate the contribution of park-based activities and self-reported park-based PA levels to overall PA in the context of high-quality, renovated urban park spaces. These findings underscore the important role of renovated urban park spaces in fostering equitable PA opportunities within underserved minority neighborhoods that may lack access to infrastructure for active living. Park improvement initiatives such as the CPI offer a promising answer to the issue of low rates of park use and park-based PA that have been previously documented among parks situated in low-income neighborhoods [9, 33, 34].

Our findings on racial/ethnic and sex differences in participation in various activities at parks align with previous research on the topic in the field of leisure sciences [33]. Latino/as in our sample reported socializing during past-month visits to their renovated study park more than any other racial/ethnic group. In previous studies, Latino/as have expressed preferences for informal social activities in parks, tending to visit parks in groups of three or more people [16, 18, 19, 33, 35, 36]. Black individuals in our sample reported the second highest prevalence of engaging in social behaviors during past-month renovated park visits, which could reflect a preference for organized group recreational opportunities and communal experiences in parks [33, 35, 37]. In addition, Blacks and Latino/s in our sample were more likely to report taking children to the playground/park compared to other groups, suggesting a more family-centered orientation to park use among these groups [38].

We also observed significant sex differences in participation in activities at renovated parks. Women were significantly more likely than men to report taking children to the playground/park, while men were significantly more likely than women to report exercising at the park. We also found that Black and White men reported significantly higher park-based PA levels compared to their female counterparts. These patterns are consistent with prior research indicating that women more often engage in caregiving roles during park visits, such as accompanying children to playgrounds, whereas men are more likely to use parks for individual exercise or leisure activities [20, 21]. Observed sex-based differences may also reflect broader societal and cultural influences. Men, regardless of race, tend to have higher levels of PA in public spaces compared to women, potentially due to differences in perceived safety, time availability, and cultural norms surrounding outdoor recreation [39].

It is apparent from our findings that improved park spaces are not a “one-size-fits-all” solution for promoting PA in underserved areas. Policies and park programming tailored to the unique needs and preferences of different demographic groups may be needed to fully activate renovated park spaces and advance health equity. Although the CPI aimed to make renovated parks more inclusive across demographic groups by incorporating diverse features such as picnic areas, walking paths, ball courts, shaded seating areas, playgrounds, and adult fitness equipment, these elements alone may not be sufficient to encourage park use and park-based PA among all groups [40]. Variation in the contribution of specific park-based activities to overall PA across demographic groups highlights opportunities for tailored programming, particularly for groups less likely to achieve recommended weekly PA levels, such as women and minority groups [41]. For example, among Latino/as in our sample, exercising and socializing at renovated parks were the strongest positive correlates of overall PA, suggesting that park-based programs incorporating group exercise activities with family and friends may be particularly effective at promoting PA in this population [18, 42]. Among non-Latino Black individuals, walking at renovated parks showed the strongest association with overall PA, followed by exercising. This pattern suggests that walking groups or park-based fitness classes could be beneficial, particularly among Black women [43], who in our sample reported lower PA levels during CPI park visits compared with Black men. For non-Latino Whites, taking children to the playground/park was negatively associated with overall PA, indicating that park-based PA interventions for White parents (as well as parents in other racial/ethnic groups) may benefit from the provision of childcare or colocation of childcare facilities near the renovated parks [44, 45]. Recognizing the role of park activity patterns and preferences, as well as barriers to park-based PA, can help inform more effective park-based health promotion strategies aimed at reducing racial/ethnic and sex disparities in PA.

Community engagement is another vital consideration in leveraging renovated park spaces to improve PA and other health outcomes. The data for the analyses reported in this paper were collected as part of the baseline assessment for the SPARCS project – an ongoing community-engaged intervention study designed to activate recently renovated CPI park spaces through tailored programming that reflects the needs and preferences of neighborhood residents [27]. Prior research has demonstrated the value of community engagement and co-design in maximizing parks’ impact on PA and community health [46, 47]. For example, in a 2013 study across 50 Los Angeles parks, Cohen et al. showed that modest discretionary funds paired with participatory decision-making by park directors and advisory boards led to successful community-prioritized park enhancements such as signage, outreach events, and new group activities, as well as significant increases in park-based PA at intervention sites compared to control sites [46]. Similarly, in a 2015 study, Patton-López et al. implemented a resident-led design charrette at a park serving a low-income community in Oregon which resulted in the installation of play structures and climbing features that reflected community preferences while strengthening local relationships and civic capacity, leading to a greater variety of activities observed among children and adolescents visiting the park [47]. Grounded in this tradition, the ongoing SPARCS intervention is empowering local groups to co-develop and lead programming (such as salsa dancing classes, sculpture workshops, and pickup basketball games) that builds on community strengths and culture [27]. Given our findings on demographic differences in the role of renovated parks in fostering park-based PA, future park renovations and programming initiatives should prioritize such community-driven strategies to optimize the use of high-quality park spaces in ways that equitably enhance health and well-being across diverse groups.

A key strength of our study was its large and diverse sample, which allowed for a nuanced examination of park-based PA behaviors across various demographic groups and strengthened the generalizability of our findings by capturing real-world variability in PA patterns. The novel exploration of specific park-based activities and their contributions to overall PA further enhances our understanding of how different populations engage with high-quality urban green spaces. However, several limitations should be acknowledged. Measures of park use, activities, and usual PA levels during visits to renovated parks were based on self-reported past-month recall, which may not accurately capture actual participation in activities or true PA intensity. Additionally, overall PA was estimated using the IPAQ, a tool known to overestimate PA relative to objective measurement tools like accelerometry [48]. Future research could incorporate wearable technology to obtain more precise and objective PA data in large, population-representative samples [49], and to validate associations observed using self-reported PA measures. Finally, although analyses of individual parks and specific features were beyond the scope of this study, future research using qualitative or mixed-methods approaches could provide valuable insight into how different demographic groups interact with specific features of renovated parks and which amenities most effectively support their engagement.

## Conclusions

This study highlights an important role of high-quality renovated urban park spaces in promoting PA, particularly among underserved communities that face structural barriers to active living. By identifying demographic differences in activities at renovated parks and their contributions to overall PA, our findings reinforce the need for equitable, community-driven park-based PA interventions that reflect diverse cultural preferences and engagement patterns. Future park improvement initiatives should not only focus on physical enhancements but also prioritize programming strategies tailored to the needs of specific demographic sub-groups. As cities continue investing in urban green spaces, integrating inclusive, evidence-based approaches to park design and programming can serve as a powerful tool for addressing disparities in PA and advancing public health equity.

## Supplementary Information

Additional File 1.

Additional File 2.

## Data Availability

The datasets used and/or analyzed during the current study are available from the corresponding author on reasonable request.

## Abbreviation

PA: Physical activity
US: United States
NYC: New York City
CPI: Community Parks Initiative
IPAQ: International Physical Activity Questionnaire
SPARCS: Supporting Parks and Revitalizing Communities Study
MET: Metabolic equivalent of task
