# Supplementary material for: Contributions of park-based activities to overall physical activity among adults living near recently renovated parks in low-income New York City neighborhoods: variations by race/ethnicity and sex
Source: Int J Behav Nutr Phys Act. 2025 Nov 12;22:143. doi: 10.1186/s12966-025-01838-0 (PMC12613851; doi:10.1186/s12966-025-01838-0)
Supplement: Supplementary file 1 — Additional File 1. [file 12966_2025_1838_MOESM1_ESM.docx]

STROBE Checklist for “Contributions of park-based activities to overall physical activity among adults living near recently renovated parks in low-income New York City neighborhoods: Variations by race/ethnicity and sex” by Rachel L. Thompson, Luis David Olivera León, Houlin Hong, Justine Maffei, Katarzyna E. Wyka, and Terry T.-K. Huang

|  | | | Item No. | Recommendation | Page  No. | | | Relevant text from manuscript |
| --- | --- | --- | --- | --- | --- | --- | --- | --- |
| **Title and abstract** | | | 1 | (*a*) Indicate the study’s design with a commonly used term in the title or the abstract | 1-2 | | | In this ***cross-sectional*** study, we examined associations between park-based activities and overall PA among adults living near recently renovated parks in lower-income New York City (NYC) neighborhoods, with particular attention to differences by race/ethnicity and sex. |
|  |  |  |  | (*b*) Provide in the abstract an informative and balanced summary of what was done and what was found | 1-2 | | | See ***Abstract*** |
| Introduction | | | | | | | |  |
| Background/rationale | | | 2 | Explain the scientific background and rationale for the investigation being reported | 3-5 | | | See ***Background*** |
| Objectives | | | 3 | State specific objectives, including any prespecified hypotheses | 5 | | | In this study, we sought to examine how park-based activities and park-based PA at high-quality, recently renovated urban parks contribute to overall PA across diverse sociodemographic groups. |
| Methods | | | | | | | |  |
| Study design | | | 4 | Present key elements of study design early in the paper | 5-6 | | | See ***Methods > Setting*** and ***Methods > Survey data collection*** |
| Setting | | | 5 | Describe the setting, locations, and relevant dates, including periods of recruitment, exposure, follow-up, and data collection | 5-6 | | | See ***Methods > Setting*** and ***Methods > Survey data collection*** |
| Participants | | | 6 | (*a*) *Cohort study*—Give the eligibility criteria, and the sources and methods of selection of participants. Describe methods of follow-up  *Case-control study*—Give the eligibility criteria, and the sources and methods of case ascertainment and control selection. Give the rationale for the choice of cases and controls  *Cross-sectional study*—Give the eligibility criteria, and the sources and methods of selection of participants | 6 | | | Participants were recruited from the residential areas located within a 0.5-mile radius of each of the eight included CPI parks. Recruitment was conducted by the Consensus Strategies polling firm (Waltham, MA) via email and text message. We employed a stratified random sampling approach with a minimum of 25 participants per stratum per park catchment area. Stratification was based on sex, age, race/ethnicity, and education level, with enrollment targets chosen to reflect the demographic composition of each park’s surrounding neighborhood. |
|  |  |  |  | (*b*) *Cohort study*—For matched studies, give matching criteria and number of exposed and unexposed  *Case-control study*—For matched studies, give matching criteria and the number of controls per case | N/A | | | N/A |
| Variables | | | 7 | Clearly define all outcomes, exposures, predictors, potential confounders, and effect modifiers. Give diagnostic criteria, if applicable | 6-8 | | | See ***Methods > Measures***  Outcome = ***MET-Minutes of PA***  Exposures/predictors = ***park-based activities and park-based PA***  Potential confounders = ***education level, annual household income, age, employment status, frequency of study park use, frequency of other park use, and study site***  Effect modifiers = ***race/ethnicity*** and ***sex*** |
| Data sources/ measurement | | | 8* | For each variable of interest, give sources of data and details of methods of assessment (measurement). Describe comparability of assessment methods if there is more than one group | 6-8 | | | See ***Methods > Measures*** |
| Bias | | | 9 | Describe any efforts to address potential sources of bias | 6 | | | See ***Methods > Survey data collection*** and ***Methods > Statistical Analysis*** |
| Study size | | | 10 | Explain how the study size was arrived at | 6 | | | See ***Methods > Survey data collection*** |
| Quantitative variables | | 11 | | Explain how quantitative variables were handled in the analyses. If applicable, describe which groupings were chosen and why | 6-7, 9 | | See ***Methods > Measures > Outcome – MET-minutes of PA***  And ***Methods > Statistical Analysis*** Prior to analysis, the outcome variable (MET-minutes) was natural log-transformed after adding a small amount (+30 minutes) to each observation to account for zero values. | |
| Statistical methods | | 12 | | (*a*) Describe all statistical methods, including those used to control for confounding | 8-9 | | See ***Methods > Statistical analysis*** | |
|  |  |  |  | (*b*) Describe any methods used to examine subgroups and interactions | 9 | | Linear regression models were used to characterize the contribution of participation in park-based activities to overall PA in the overall sample of past-month study park users and separately by race/ethnicity, sex, and the combination of race/ethnicity and sex. | |
|  |  |  |  | (*c*) Explain how missing data were addressed | N/A | | N/A (no missing data) | |
|  |  |  |  | (*d*) *Cohort study*—If applicable, explain how loss to follow-up was addressed  *Case-control study*—If applicable, explain how matching of cases and controls was addressed  *Cross-sectional study*—If applicable, describe analytical methods taking account of sampling strategy | N/A | | N/A | |
|  |  |  |  | (*e*) Describe any sensitivity analyses | N/A | | N/A | |
| Results | | | | | | | | |
| Participants | | 13* | | (a) Report numbers of individuals at each stage of study—eg numbers potentially eligible, examined for eligibility, confirmed eligible, included in the study, completing follow-up, and analysed | 9 | | Out of 2,067 individuals who completed the survey, 1,336 who reported using their renovated study park at least once in the past 30 days were included in this analysis. | |
|  |  |  |  | (b) Give reasons for non-participation at each stage | N/A | | N/A | |
|  |  |  |  | (c) Consider use of a flow diagram | N/A | | N/A | |
| Descriptive data | | 14* | | (a) Give characteristics of study participants (eg demographic, clinical, social) and information on exposures and potential confounders | 9-12 | | See ***Results > Sample sociodemographic characteristics***; ***Results > Differences in park-based activities and park-based PA by race/ethnicity and sex among past-month renovated park users***; ***Tables 1-3*** | |
|  |  |  |  | (b) Indicate number of participants with missing data for each variable of interest | N/A | | N/A | |
|  |  |  |  | (c) *Cohort study*—Summarise follow-up time (eg, average and total amount) | N/A | | N/A | |
| Outcome data | | 15* | | *Cohort study*—Report numbers of outcome events or summary measures over time | N/A | | N/A | |
|  |  |  |  | *Case-control study—*Report numbers in each exposure category, or summary measures of exposure | N/A | | N/A | |
|  |  |  |  | *Cross-sectional study—*Report numbers of outcome events or summary measures | 26 | | See ***Table 1*** | |
| Main results | | 16 | | (*a*) Give unadjusted estimates and, if applicable, confounder-adjusted estimates and their precision (eg, 95% confidence interval). Make clear which confounders were adjusted for and why they were included | 13-14 | | See ***Results > Differences in contributions of park-based activities to overall PA among past-month renovated park users by race/ethnicity and sex***; ***Tables 4-5*** | |
|  |  |  |  | (*b*) Report category boundaries when continuous variables were categorized | N/A | | N/A | |
|  |  |  |  | (*c*) If relevant, consider translating estimates of relative risk into absolute risk for a meaningful time period | N/A | | N/A | |
| Other analyses | 17 | | Report other analyses done—eg analyses of subgroups and interactions, and sensitivity analyses | | 12-13 | See ***Results > Contributions of self-reported usual park-based PA level to overall PA among past-month renovated park users***; ***Additional File 2 – Tables 2-3*** | | |
| Discussion | | | | | | | | |
| Key results | 18 | | Summarise key results with reference to study objectives | | 14-15 | See first paragraph of ***Discussion*** | | |
| Limitations | 19 | | Discuss limitations of the study, taking into account sources of potential bias or imprecision. Discuss both direction and magnitude of any potential bias | | 17-18 | See last paragraph of ***Discussion*** | | |
| Interpretation | 20 | | Give a cautious overall interpretation of results considering objectives, limitations, multiplicity of analyses, results from similar studies, and other relevant evidence | | 14-18 | See ***Discussion*** | | |
| Generalisability | 21 | | Discuss the generalisability (external validity) of the study results | | 17 | A key strength of our study is its large and diverse sample, which allows for a nuanced examination of park-based PA behaviors across various demographic groups and strengthens the generalizability of our findings by capturing real-world variability in PA patterns. | | |
| Other information | | |  | | | | | |
| Funding | 22 | | Give the source of funding and the role of the funders for the present study and, if applicable, for the original study on which the present article is based | | 19 | This study was supported in part by funding from the National Institutes of Health (R01MD018209) and the Centers for Disease Control and Prevention (U48DP006396). The funders were not involved in the conceptualization, design, data collection, analysis, decision to publish or preparation of this manuscript. The content is solely the responsibility of the authors and does not necessarily represent the official views of the funding agencies. | | |

*Give information separately for cases and controls in case-control studies and, if applicable, for exposed and unexposed groups in cohort and cross-sectional studies.

**Note:** An Explanation and Elaboration article discusses each checklist item and gives methodological background and published examples of transparent reporting. The STROBE checklist is best used in conjunction with this article (freely available on the Web sites of PLoS Medicine at http://www.plosmedicine.org/, Annals of Internal Medicine at http://www.annals.org/, and Epidemiology at http://www.epidem.com/). Information on the STROBE Initiative is available at www.strobe-statement.org.
