# Supplementary material for: Contributions of park-based activities to overall physical activity among adults living near recently renovated parks in low-income New York City neighborhoods: variations by race/ethnicity and sex
Source: Int J Behav Nutr Phys Act. 2025 Nov 12;22:143. doi: 10.1186/s12966-025-01838-0 (PMC12613851; doi:10.1186/s12966-025-01838-0)
Supplement: Supplementary file 2 — Additional File 2. [file 12966_2025_1838_MOESM2_ESM.docx]

**Contributions of park-based activities to overall physical activity among adults living near recently renovated parks in low-income New York City neighborhoods: Variations by race/ethnicity and sex**

Supplemental Material

Authors: Rachel L. Thompson, Luis David Olivera León, Houlin Hong, Justine Maffei, Katarzyna E. Wyka, and Terry T.-K. Huang

**Table of Contents**

**Supplemental Table 1. Characteristics of the complete survey sample stratified by past 30-day study park use**

**Supplemental Table 2. Associations of self-reported usual PA level during past 30-day visits to the study park and MET-minutes of PA in the overall survey sample and by race/ethnicity**

**Supplemental Table 3. Associations of self-reported usual PA level during past 30-day visits to the study park and MET-minutes of PA in the overall survey sample and by sex and race/ethnicity**

**Supplemental Table 1. Characteristics of the complete survey sample and differences by past 30-day study park use**

|  | **Complete Sample**  n = 2,067^a^ | **Visited study park in the past 30 days**  n = 1,336 (65%)^a^ | **Did not visit study park in the past 30 days**  n = 731 (35%)^a^ | p-value^b^ |
| --- | --- | --- | --- | --- |
| **Race/Ethnicity** |  |  |  | 0.006 |
| Latino/a | 705 (34%) | 475 (36%) | 230 (31%) |  |
| Non-Latino/a Black | 639 (31%) | 378 (28%) | 261 (36%) |  |
| Non-Latino/a White | 441 (21%) | 291 (22%) | 150 (21%) |  |
| Other | 282 (14%) | 192 (14%) | 90 (12%) |  |
| **Sex** |  |  |  | 0.013 |
| Female | 1376 (67%) | 864 (65%) | 512 (70%) |  |
| Male | 691 (33%) | 472 (35%) | 219 (30%) |  |
| **Education Level** |  |  |  | 0.749 |
| Some college or more | 1595 (77%) | 1028 (77%) | 567 (78%) |  |
| High school graduate or less | 472 (23%) | 308 (23%) | 164 (22%) |  |
| **Annual Household Income** |  |  |  | 0.409 |
| <$25,000 | 514 (25%) | 321 (24%) | 193 (26%) |  |
| $25,000-$75,000 | 789 (38%) | 510 (38%) | 279 (38%) |  |
| ≥$75,000 | 764 (37%) | 505 (38%) | 259 (35%) |  |
| **Age** |  |  |  | 0.003 |
| 18-35y | 651 (31%) | 441 (33%) | 210 (29%) |  |
| 36-59y | 1082 (52%) | 705 (53%) | 377 (52%) |  |
| >60y | 334 (16%) | 190 (14%) | 144 (20%) |  |
| **Employment Status** |  |  |  | 0.075 |
| Employed or self-employed | 1364 (66%) | 898 (67%) | 466 (64%) |  |
| Not employed | 492 (24%) | 316 (24%) | 176 (24%) |  |
| Retired | 211 (10%) | 122 (9%) | 39 (12%) |  |
| **Borough** |  |  |  | 0.056 |
| Bronx | 509 (25%) | 319 (24%) | 190 (26%) |  |
| Brooklyn | 514 (25%) | 328 (25%) | 186 (25%) |  |
| Manhattan | 528 (26%) | 330 (25%) | 198 (27%) |  |
| Queens | 516 (25%) | 359 (27%) | 157 (21%) |  |
| **MET-Minutes of Physical Activity** |  |  |  | 0.001 |
|  | 1,335 [495-2,820] | 1,386 [584-2,853] | 1,173 [396-2,772] |  |
| ^a^n (%); median [IQR]  ^b^Pearson’s Chi-squared test; Wilcoxon rank sum test  Abbreviations – CPI: Community Parks Initiative; NYC: New York City | | | | |

**Supplemental Table 2. Associations of self-reported usual PA level during past 30-day visits to the study park and MET-minutes of PA in the overall survey sample and by race/ethnicity**

|  |  | **Unadjusted Models** | | |  | **Adjusted Models^b^** | |
| --- | --- | --- | --- | --- | --- | --- | --- |
| **Subgroup** | **Usual PA Level During Visits to Study Park in Past 30 Days** | **e^β^ (95% CI)^a^** | **p-value** | **Model R^2^** |  | **e^β^ (95% CI)^a^** | **p-value** |
| **Overall**  **(n = 1,336)** | Mostly moderate-to-vigorous activities | 2.09 (1.76, 2.48) | <.001 | 0.052 |  | 1.95 (1.64, 2.32) | <.001 |
|  | Mostly light activities | 1.64 (1.40, 1.92) | <.001 |  |  | 1.51 (1.29, 1.76) | <.001 |
|  | Mostly sitting activities | *Reference Group* |  |  |  | *Reference Group* |  |
| **Latino/a**  **(n = 475)** | Mostly moderate-to-vigorous activities | 2.09 (1.57, 2.78) | <.001 | 0.053 |  | 2.06 (1.55, 2.76) | <.001 |
|  | Mostly light activities | 1.64 (1.25, 2.15) | <.001 |  |  | 1.57 (1.20, 2.06) | 0.001 |
|  | Mostly sitting activities | *Reference Group* |  |  |  | *Reference Group* |  |
| **Non-Latino/a Black**  **(n = 378)** | Mostly moderate-to-vigorous activities | 2.20 (1.58, 3.06) | <.001 | 0.057 |  | 2.10 (1.50, 2.95) | <.001 |
|  | Mostly light activities | 1.59 (1.18, 2.16) | 0.003 |  |  | 1.50 (1.09, 2.07) | 0.012 |
|  | Mostly sitting activities | *Reference Group* |  |  |  | *Reference Group* |  |
| **Non-Latino/a White**  **(n = 291)** | Mostly moderate-to-vigorous activities | 1.58 (1.12, 2.22) | 0.009 | 0.026 |  | 1.44 (1.01, 2.06) | 0.043 |
|  | Mostly light activities | 1.39 (1.02, 1.89) | 0.035 |  |  | 1.21 (0.87, 1.66) | 0.252 |
|  | Mostly sitting activities | *Reference Group* |  |  |  | *Reference Group* |  |
| **Other**  **(n = 192)** | Mostly moderate-to-vigorous activities | 2.61 (1.60, 4.23) | <.001 | 0.084 |  | 2.39 (1.41, 4.04) | 0.001 |
|  | Mostly light activities | 1.89 (1.27, 2.82) | 0.002 |  |  | 1.79 (1.16, 2.75) | 0.009 |
|  | Mostly sitting activities | *Reference Group* |  |  |  | *Reference Group* |  |

^a^Obtained from linear regression models with the outcome log(MET-minutes of past-week PA + 30).

^b^Additionally adjusted for sex, education level, annual household income, age, employment status, frequency of study park use, frequency of other park use, and study site. The overall model was also adjusted for race/ethnicity.

Abbreviations – PA: physical activity; MET: metabolic equivalent of task

**Supplemental Table 3. Associations of self-reported usual PA level during past 30-day visits to the study park and MET-minutes of PA in the overall survey sample and by sex and race/ethnicity**

|  |  | **Unadjusted Models** | | |  | **Adjusted Models^b^** | |
| --- | --- | --- | --- | --- | --- | --- | --- |
| **Subgroup** | **Usual PA Level During Visits to Study Park in Past 30 Days** | **e^β^ (95% CI)^a^** | **p-value** | **Model R^2^** |  | **e^β^ (95% CI)^a^** | **p-value** |
| **Female**  **(n = 864)** | Mostly moderate-to-vigorous activities | 1.88 (1.52, 2.34) | <.001 | 0.041 |  | 1.79 (1.44, 2.22) | <.001 |
|  | Mostly light activities | 1.60 (1.32, 1.93) | <.001 |  |  | 1.44 (1.19, 1.75) | <.001 |
|  | Mostly sitting activities | *Reference Group* |  |  |  | *Reference Group* |  |
| **Male**  **(n = 472)** | Mostly moderate-to-vigorous activities | 2.44 (1.83, 3.25) | <.001 | 0.075 |  | 2.20 (1.65, 2.94) | <.001 |
|  | Mostly light activities | 1.74 (1.32, 2.28) | <.001 |  |  | 1.60 (1.22, 2.10) | 0.001 |
|  | Mostly sitting activities | *Reference Group* |  |  |  | *Reference Group* |  |
| **Latina Female**  **(n = 328)** | Mostly moderate-to-vigorous activities | 1.90 (1.36, 2.67) | <.001 | 0.045 |  | 1.93 (1.37, 2.72) | <.001 |
|  | Mostly light activities | 1.63 (1.18, 2.25) | 0.003 |  |  | 1.58 (1.14, 2.18) | 0.006 |
|  | Mostly sitting activities | *Reference Group* |  |  |  | *Reference Group* |  |
| **Latino Male**  **(n = 147)** | Mostly moderate-to-vigorous activities | 2.69 (1.55, 4.67) | 0.001 | 0.081 |  | 2.40 (1.35, 4.26) | 0.003 |
|  | Mostly light activities | 1.67 (1.01, 2.74) | 0.044 |  |  | 1.45 (0.87, 2.41) | 0.147 |
|  | Mostly sitting activities | *Reference Group* |  |  |  | *Reference Group* |  |
| **Non-Latina Black Female**  **(n = 276)** | Mostly moderate-to-vigorous activities | 1.75 (1.17, 2.62) | 0.006 | 0.031 |  | 1.74 (1.15, 2.62) | 0.008 |
|  | Mostly light activities | 1.52 (1.07, 2.14) | 0.019 |  |  | 1.47 (1.02, 2.13) | 0.041 |
|  | Mostly sitting activities | *Reference Group* |  |  |  | *Reference Group* |  |
| **Non-Latino Black Male**  **(n = 102)** | Mostly moderate-to-vigorous activities | 3.30 (1.81, 6.00) | <.001 | 0.137 |  | 3.85 (2.00, 7.40) | <.001 |
|  | Mostly light activities | 1.83 (0.94, 3.56) | 0.077 |  |  | 2.14 (1.04, 4.38) | 0.038 |
|  | Mostly sitting activities | *Reference Group* |  |  |  | *Reference Group* |  |
| **Non-Latina White Female**  **(n = 151)** | Mostly moderate-to-vigorous activities | 1.48 (0.87, 2.52) | 0.151 | 0.015 |  | 1.23 (0.68, 2.21) | 0.489 |
|  | Mostly light activities | 1.27 (0.83, 1.95) | 0.266 |  |  | 1.00 (0.61, 1.62) | 0.995 |
|  | Mostly sitting activities | *Reference Group* |  |  |  | *Reference Group* |  |
| **Non-Latino White Male**  **(n = 140)** | Mostly moderate-to-vigorous activities | 1.63 (1.04, 2.56) | 0.034 | 0.039 |  | 1.56 (0.97, 2.52) | 0.065 |
|  | Mostly light activities | 1.60 (1.02, 2.49) | 0.04 |  |  | 1.40 (0.88, 2.23) | 0.152 |
|  | Mostly sitting activities | *Reference Group* |  |  |  | *Reference Group* |  |
| **Other Female**  **(n = 109)** | Mostly moderate-to-vigorous activities | 2.82 (1.54, 5.15) | 0.001 | 0.107 |  | 2.72 (1.41, 5.25) | 0.003 |
|  | Mostly light activities | 1.90 (1.14, 3.16) | 0.014 |  |  | 1.69 (0.96, 2.97) | 0.068 |
|  | Mostly sitting activities | *Reference Group* |  |  |  | *Reference Group* |  |
| **Other Male**  **(n = 83)** | Mostly moderate-to-vigorous activities | 2.25 (0.98, 5.16) | 0.054 | 0.052 |  | 1.42 (0.55, 3.66) | 0.464 |
|  | Mostly light activities | 1.76 (0.90, 3.43) | 0.098 |  |  | 1.65 (0.76, 3.56) | 0.198 |
|  | Mostly sitting activities | *Reference Group* |  |  |  | *Reference Group* |  |

^a^Obtained from linear regression models with the outcome log(MET-minutes of past-week PA + 30).

^b^Additionally adjusted for education level, annual household income, age, employment status, frequency of study park use, frequency of other park use, and study site. The overall models for males and females were also adjusted for race/ethnicity.

Abbreviations – PA: physical activity; MET: metabolic equivalent of task
